# Botulinum toxin-induced facial muscle paralysis affects amygdala responses to the perception of emotional expressions: preliminary findings from an A-B-A design

**DOI:** 10.1186/2045-5380-4-11

**Published:** 2014-10-31

**Authors:** M Justin Kim, Maital Neta, F Caroline Davis, Erika J Ruberry, Diana Dinescu, Todd F Heatherton, Mitchell A Stotland, Paul J Whalen

**Affiliations:** 1 Department of Psychological and Brain Sciences, Dartmouth College, 6207 Moore Hall, Hanover, NH 03755, USA; 2 Department of Psychology, B84 East Stadium, University of Nebraska-Lincoln, Lincoln, NE 68588-0156, USA; 3 US Army Natick Soldier Research Development and Engineering Center, Cognitive Science Research Team, 10 Kansas St., Natick, MA 01760, USA; 4 Department of Psychology, Tufts University, 490 Boston Avenue, Medford, MA 02155, USA; 5 Department of Psychology, University of Washington, 119A Guthrie Hall, Seattle, WA 98195, USA; 6 Department of Psychology, University of Virginia, 102 Gilmer Hall, Charlottesville, VA 22904, USA; 7 Department of Surgery (Plastic) and Pediatrics, Geisel School of Medicine at Dartmouth, Dartmouth-Hitchcock Medical Center, Lebanon, NH 03756, USA; 8 Department of Surgery (Plastic and Craniofacial), Sidra Research and Medical Center, Doha, Qatar

**Keywords:** Amygdala, Botulinum toxin, Corrugator supercilii, Facial feedback hypothesis

## Abstract

**Background:**

It has long been suggested that feedback signals from facial muscles influence emotional experience. The recent surge in use of botulinum toxin (BTX) to induce temporary muscle paralysis offers a unique opportunity to directly test this “facial feedback hypothesis.” Previous research shows that the lack of facial muscle feedback due to BTX-induced paralysis influences subjective reports of emotional experience, as well as brain activity associated with the imitation of emotional facial expressions. However, it remains to be seen whether facial muscle paralysis affects brain activity, especially the amygdala, which is known to be responsive to the perception of emotion in others. Further, it is unknown whether these neural changes are permanent or whether they revert to their original state after the effects of BTX have subsided. The present study sought to address these questions by using functional magnetic resonance imaging to measure neural responses to angry and happy facial expressions in the presence or absence of facial paralysis.

**Results:**

Consistent with previous research, amygdala activity was greater in response to angry compared to happy faces before BTX treatment. As predicted, amygdala activity in response to angry faces was attenuated when the corrugator/procerus muscles were paralyzed via BTX injection but then returned to its original state after the effects of BTX subsided. This preliminary study comprises a small sample size and no placebo condition; however, the A-B-A design affords the present sample to serve as its own control.

**Conclusions:**

The current demonstration that amygdala responses to facial expressions were influenced by facial muscle paralysis offers direct neural support for the facial feedback hypothesis. Specifically, the present findings offer preliminary causal evidence that amygdala activity is sensitive to facial feedback during the perception of the facial expressions of others. More broadly, these data confirm the utility of using BTX to address the effect of facial feedback on neural responses associated with the *perception*, in addition to the experience or expression of emotion.

## Background

It has long been suggested that feedback signals from facial muscle activity associated with forming facial expressions are critical to emotional experience [1,2]. This notion has developed into what we now know as the facial feedback hypothesis [3]. According to this hypothesis, our brain receives afferent feedback signals from facial muscles that significantly influence how we process and experience emotion [4]. Importantly, a number of psychological experiments have shown support for the facial feedback hypothesis [5-9].

In a classic demonstration by Strack and colleagues, participants judged the funniness of cartoons while unknowingly contracting their zygomaticus (the facial muscle used during smiling) by holding a pen in their mouths [9]. This subtle manipulation influenced their perception of the cartoons; such that they found the cartoons to be funnier when contracting the zygomaticus muscles compared to a control condition where the zygomaticus muscles were not contracted. This study offered clear behavioral evidence that feedback signals from the facial muscles influence our emotional experiences.

Seminal studies such as this would be strengthened by additional work seeking to establish a direct causal relationship between facial feedback and emotional phenomena. Though Strack and colleagues convincingly employed methods that protected against demand characteristics (i.e., participants ostensibly were not aware of the fact that they were being asked to smile) [9], there are additional considerations when asking participants to actively alter their facial muscle contractions. Specifically, intentional alteration of facial muscle activity calls for novel efferent signals to be sent to the facial muscles, which might influence how we experience emotion independent of any change in the afferent facial feedback signals to the brain. To put it another way, this method does not allow us to distinguish between whether the observed outcomes are due to a change in brain activity that is responsible for producing the emotional facial expression (efferent) or a change in facial feedback signals to the brain (afferent) [10,11]. Thus, it would be ideal to leave the efferent brain signals intact while selectively shutting down the afferent facial muscle signals.

Recent widespread cosmetic use of botulinum toxin (BTX) type-A has allowed researchers to perform exactly this separation of afferent and efferent signals, making it possible to directly test causal factors related to facial feedback. BTX induces a temporary paralysis of the muscles at the injection site by inhibiting the release of the neurotransmitter acetylcholine at the muscle nerves [12]. By injecting BTX into the muscles that we use to make emotional facial expressions, a “reversible lesion” of target facial muscles can be temporarily created. This effectively “severs” afferent feedback signals from these muscles while leaving the efferent signals intact, thus allowing us to study emotional experience and processing devoid of facial muscle feedback.

The corrugator supercilii—an important component of the glabellar muscles (the “frown muscles” between the eyebrows) along with the procerus [13]—has been implicated in the experience and processing of negative emotions. One prototypical feature of an angry facial expression is that the medial portions of the eyebrows are pulled down using the corrugator supercilii muscles [14]. Using facial electromyography (EMG), researchers have shown that corrugator activity is selectively potentiated when viewing photos of angry facial expressions [15-17]. A similar pattern of corrugator activity is found in response to negative affective pictures [18-20] and sounds [20].

Based on this established link between corrugator activity and negative emotion, a number of recent studies have used BTX to directly test the facial feedback hypothesis and found that feedback from facial muscles influenced subjective experience of emotion and emotional language processing [10,21]. Other studies suggest that BTX injections mitigate depression symptoms and help elevate mood in major depressive disorder patients [22,23]. Functional neuroimaging studies are also beginning to explore the relationship between facial feedback and responses to emotional stimuli. The first such study assessed the effect of BTX on the ability to imitate emotional expressions [24]. Amygdala activity, and its coupling with brain stem activity, was diminished when the corrugator muscle was paralyzed with BTX as participants attempted to imitate angry facial expressions. Taken together, these data suggest that our emotional experiences as well as brain activity to emotional stimuli can be influenced by feedback signals from the facial muscles.

The present study sought to determine whether the activity of the amygdala—a brain region within the medial temporal lobe that is known to be sensitive to facial expressions of emotion, including anger [25]—in response to angry facial expressions could be manipulated by BTX-induced glabellar (i.e., corrugator/procerus) muscle paralysis. Critically, when the effect of BTX injection subsides, would amygdala activity in response to angry facial expressions be restored? If so, this would provide strong evidence in favor of the facial feedback hypothesis and shed further light on its underlying neural mechanism. To this end, we used functional magnetic resonance imaging (fMRI) in an A-B-A design to assess neural responses to facial expressions a total of three times: prior to BTX treatment, shortly after BTX treatment (i.e., during corrugator/procerus paralysis), and after the effects of BTX had subsided. We hypothesized that amygdala activity would be greater to angry compared to happy facial expressions prior to BTX administration, that these signal increases to angry expressions would be mitigated by BTX, and that they would return to their pre-injection state (i.e., relatively enhanced amygdala responses to angry vs. happy faces) after BTX had subsided.

## Methods

### Participants

Initially, ten female volunteers were recruited through the Department of Plastic Surgery at Dartmouth Hitchcock Medical Center. Our study sample was limited to women for two reasons. First, reliable dosing is best obtained in females, as men have a larger glabellar muscle mass and require variable dosing. Second, the vast majority of BTX patients are women—for example, in 2013, women received 94% of 6.3 million BTX treatments in the United States [26]. Thus, we chose to focus on a female study sample. Of the ten volunteers, three did not return for at least one of the subsequent sessions, and thus, data reported here are from seven volunteers between the ages of 35–44 (mean age 40.43 ± 3.69 years). All participants had corrected-to-normal vision and were right handed. The current study was approved by the Committee for the Protection of Human Subjects at Dartmouth College, and written informed consent was obtained from each subject prior to the experiment.

### BTX injection

None of the participants had received BTX treatments prior to the current study. All participants were injected with botulinum toxin type-A a total of five times during a single visit; twice in the corrugator supercilii on each side and once in the procerus (a vertically oriented, midline muscle which pulls the medial ends of the eyebrows downward). The BTX was diluted as 100 units botulinum toxin/2 ccs non-preserved injectable saline. The volume per injection was 0.1 cc/5 units, for a total dose of 25 units. All participants received the injection 2–5 weeks after the first experimental session and then returned to the lab 3–6 weeks after the first session for a second experimental session. Finally, participants were instructed to return at least 9 months after the injection, a time known to be sufficient for the effect of BTX to dissipate [13,27]. Our participants returned for their third session an average of 54 (range 37–63) weeks after their initial BTX injection.

### Experimental paradigm

Participants were asked to participate in an fMRI scanning session at each of the three time points. During fMRI, each subject viewed a series of angry, happy, and surprised facial expressions consisting of 18 identities (9 males and 9 females), which were selected from a standardized set (NimStim) [28]. The order of presentation of all faces was randomized for each run. All of the stimuli were back-projected (Panasonic PT-D4000U DLP) onto the center of a screen, which the participants viewed using a mirror that was mounted on the head coil.

During each trial, photos of faces with angry, happy, and surprised expressions were presented for 17, 50, and 1,000 ms, followed by a black-and-white pattern that was presented for 250 ms, which served as a retinal wipe. The length of intertrial intervals was jittered between 750 and 6,750 ms (average = 3,750 ms). Each run consisted of 54 trials (18 trials for each emotion type, presented in a pseudorandom order) lasting 4.5 total minutes, and the participants underwent three runs per scanning session. During each trial, participants were asked to report using a button box whether they thought each of the faces they saw was positive or negative in emotional valence (i.e., two-alternative forced choice paradigm). We note that all trials (correct and error in ratings) were included in the subsequent fMRI analysis independent of their responses. For our purposes here, we have collapsed the data across stimulus presentation durations because our main focus was to elicit amygdala activity to angry and happy faces, regardless of the differences in duration. Surprised faces and variable stimulus durations were included as a part of a larger study, and our goal was to have the participants work on the same tasks as our previous investigation of surprise [17]. Here, we report our preliminary findings on the effects of BTX injections into the corrugator muscle on amygdala responses to angry and happy expressions.

### Image acquisition

All participants were scanned at the Dartmouth Brain Imaging Center, using a 3.0 Tesla Philips Intera Achieva Scanner (Philips Medical Systems, Bothell, WA) equipped with a SENSE birdcage head coil. Following our standard imaging protocol that is known to maximize signal to noise ratio in the amygdala in our scanner, functional images were acquired using echo-planar T2*-weighted imaging sequence. Each volume consisted of 36 interleaved 3-mm-thick axial slices with 0.5-mm interslice gap (echo time [TE] = 35 ms, repetition time [TR] = 2,000 ms, field of view [FOV] = 240 mm, flip angle = 90°, voxel size = 3 × 3 × 3.5 mm). Anatomical T1-weighted images were collected using a high-resolution 3D magnetization-prepared rapid gradient echo sequence, with 160 contiguous 1-mm-thick sagittal slices (TE = 4.6 ms, TR =9.8 ms, FOV = 240 mm, flip angle = 8°, voxel size = 1 × 0.94 × 0.94 mm).

### fMRI data analysis

All fMRI images were processed using Statistical Parametric Mapping software (SPM5, Wellcome Department of Imaging Neuroscience, London, UK). First, functional blood-oxygen-level dependent (BOLD) data were preprocessed using slice-time correction. Then, data were preprocessed to accommodate each subject’s head movement for all six directions. We note here that none of the participants showed head movement exceeding 2.5 mm or 2.5 degrees in any direction. Spatial normalization of the functional images was performed by warping our data to fit into standard space, using the Montreal Neurological Institute (MNI)-152 template. In our final preprocessing step, normalized functional images were smoothed using a Gaussian kernel of 6-mm full width at half maximum.

At each voxel, the parameter estimates of event-related activity were fit to a general linear model. Three types of events were entered in the model—angry, happy, and surprised faces. Covariates of no interest (a session mean, a linear trend for each run, and six motion parameters derived from realignment corrections) were also accounted for in the general linear model. For the purposes of the current study, we used angry and happy faces in further analysis of variance (ANOVA) investigations. Surprised faces were also included in the experimental design for another experimental hypothesis. Since surprised faces have ambiguous valence (i.e., they can be interpreted as having either positive or negative valence), understanding of these data will require further data collection in a greater number of participants. Here, we present a 3 (Session: pre-BTX, BTX, post-BTX) × 2 (Emotion: angry, happy) voxelwise ANOVA model of the data for the negative (angry) and positive (happy) expressions, since even seven participants can offer consensus on the valence of these expressions. To accommodate the 3 × 2 design (Session × Emotion), a voxelwise ANOVA model was constructed for each subject, using linear contrasts (angry vs. baseline, happy vs. baseline) generated for the three time points (pre-BTX, BTX, and post-BTX). An implicit baseline was derived from all unmodeled events in SPM—that is, all events other than angry, happy, and surprised faces. They were subsequently entered into a random effects model, which allows population-based inferences to be made from our data [29]. Given our specific hypothesis that amygdala activity would follow an A-B-A pattern in response to angry vs. happy faces, we searched for voxels that tracked this pattern by entering a quadratic contrast vector in our ANOVA model.

Since our aim was to investigate the impact of BTX on amygdala activity specifically, we selected a significance threshold of *p* < 0.05 corrected for multiple comparisons over the amygdala volume (~4,500 mm^3^), which was defined using the Automated Anatomical Labeling atlas [30]. The significance threshold was calculated through Monte Carlo simulations, using the AlphaSim tool included with the AFNI software [31]. For all other brain regions about which we did not have *a priori* hypotheses, we imposed a statistical threshold of *p* < 0.001 (uncorrected for multiple comparisons, *k* = 10 voxels) for exploratory purposes.

## Results

### Behavioral data

**Figure 1:**
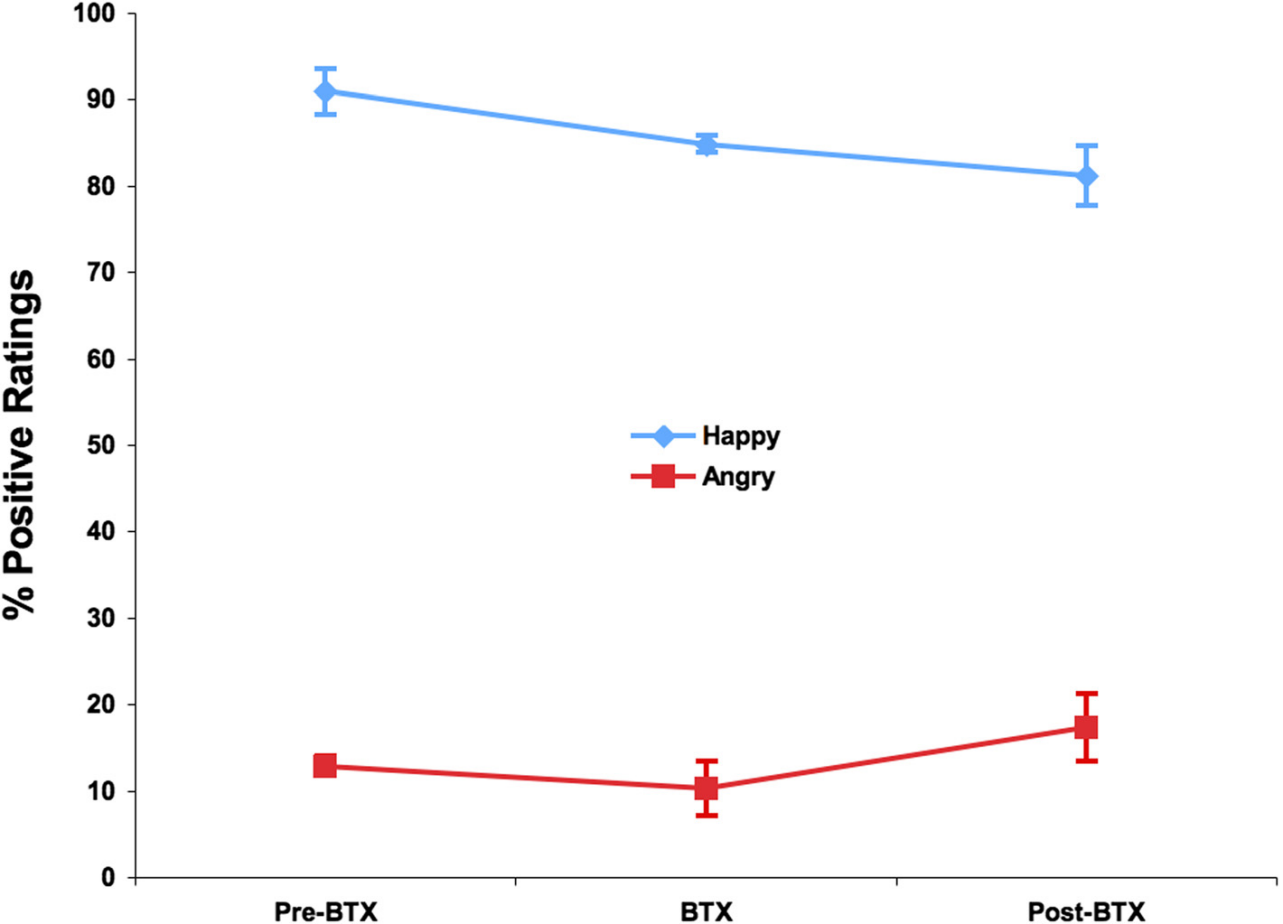
**Behavioral performance.** Behavioral data showing that angry faces are consistently rated as negative (86.5%), and happy faces are consistently rated as positive (85.7%), regardless of BTX injection. *Error bars* indicate standard error of the mean, which was computed by removing between subject-variability to account for the within-subject design [32].

A Session (pre-BTX, BTX, post-BTX) × Emotion (angry, happy) ANOVA revealed a significant main effect of Emotion (*F*_(1,6)_ = 496.73, *p* <0.001; Figure 1). As expected, post hoc pairwise comparisons revealed that across all three time points, angry faces were consistently rated more negatively than happy faces (all *p*’s < 0.001, Bonferroni corrected). We note here that accuracy was calculated by combining 50 and 1,000 ms data. Behavioral data from 17 ms were not included because past research has shown that accuracy ratings from 17 ms were not significantly different from chance [17]. Angry faces were rated as negative on 86.5% of the trials, and happy faces were rated as positive on 85.7% of the trials. The main effect of Session and the Session × Emotion interaction were not significant (all *p*’s > 0.05).

### fMRI data

**Figure 2:**
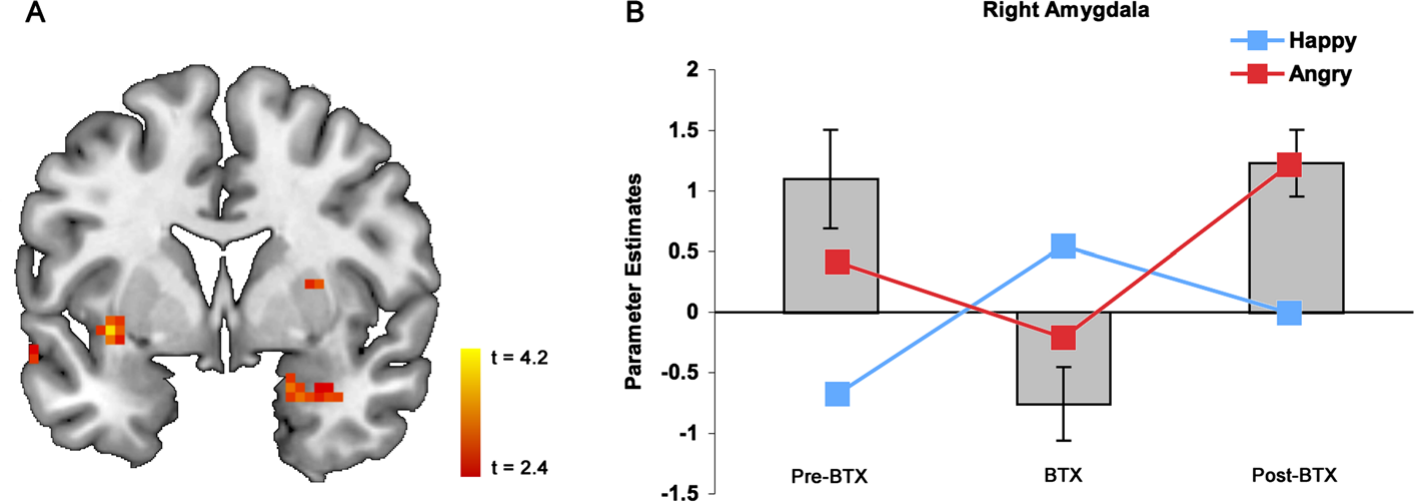
**Right amygdala activity tracks BTX-induced corrugator/procerus paralysis. (A)** Statistical map (coronal plane, *Y* = 3, *p* <0.01, *k* = 7 voxels) depicting the right amygdala (MNI 21, 3, -24; *t*_(36)_ = 3.28, *p* < 0.05 corrected, cluster size = 432 mm^3^) that corresponded to the effects of BTX. **(B)** Bar graph showing right amygdala activity to angry vs. happy faces, showing a distinctive quadratic pattern of activity (parameter estimates for angry vs. happy faces were calculated by subtracting happy vs. baseline from angry vs. baseline). *Red lines* indicate right amygdala activity to angry faces vs. baseline, and *blue lines* indicate right amygdala activity to happy faces. *Error bars* indicate standard error of the mean.

Voxelwise ANOVA results showed a significant Session × Emotion interaction characterized by a quadratic pattern of activity in the right amygdala (MNI 21, 3, -24; *t*_(36)_ = 3.28, *p* <0.05 corrected, cluster size = 432 mm^3^) in response to angry vs. happy faces (Figure 2). Specifically, this quadratic pattern was characterized by significantly increased right amygdala activity to angry vs. happy faces in pre-BTX (*p* = 0.03, one-tailed) and post-BTX (*p* = 0.04, one-tailed) conditions but no significant activity in the BTX condition. No significant main effects of Session or Emotion, as well as linear interaction effects were observed in the amygdala. No other brain regions showed this quadratic pattern of activity in response to angry vs. happy faces at the pre-determined statistical threshold.

In an attempt to examine the possibility that the observed quadratic effect was driven by amygdala activity to angry faces or happy faces compared to baseline, we further probed the right amygdala voxel cluster defined by the voxelwise ANOVA (see Figure 2A) and analyzed angry vs. baseline and happy vs. baseline separately. An ANOVA with a planned quadratic contrast revealed that right amygdala activity to angry vs. baseline (*F*_(1,6)_ = 6.08, *p* = 0.049) and happy vs. baseline (*F*_(1,6)_ =10.9, *p* = 0.016) had significant quadratic effects across sessions, showing that the aforementioned voxelwise ANOVA results were not driven by angry faces or happy faces alone (Figure 2). In fact, the quadratic pattern of amygdala activity to angry faces had a U-shaped curve, which matched the pattern observed in response to angry vs. happy faces, whereas amygdala activity to happy faces demonstrated an inverted U-shaped curve. There were no significant linear effects across sessions for either angry vs. baseline or happy vs. baseline comparisons (all *p*’s > 0.05).

## Discussion

The aim of the current study was to investigate the effects of BTX-induced corrugator/procerus paralysis on amygdala activity to angry faces by adapting an A-B-A experimental design, a powerful method that allowed us to determine the neural consequence of BTX treatment. Consistent with our hypothesis, we observed diminished amygdala activity to angry vs. happy faces when the facial muscles used to produce angry facial expressions were paralyzed. Importantly, amygdala activity was restored to its pre-BTX injection state after the effects of BTX subsided. In other words, amygdala activity in response to angry vs. happy faces was modulated in parallel with the activity state of the corrugator supercilii and the procerus. When the corrugator and procerus muscles were unaffected by BTX, greater amygdala activity to angry vs. happy faces was observed. However, when the corrugator and procerus muscles were paralyzed via BTX injection, amygdala activity in response to angry vs. happy faces was attenuated. Our finding implies that feedback signals from the corrugator supercilii and the procerus to the brain are modulating amygdala activity.

According to the facial feedback hypothesis, our emotional experience is modulated by feedback signals from the facial muscles that we use to create expressions, and this argument is supported, at least in part, by a number of psychological studies [6,8-10,21]. Temporary paralysis of facial muscles using BTX essentially cuts off all afferent feedback signals from the injection site while leaving the efferent signals untouched [12], providing researchers with an ideal setup to directly test the facial feedback hypothesis. Behavioral and psychophysiological studies utilizing these unique properties of BTX have also partially supported the facial feedback hypothesis [10,21]. Here, we use the term “partially” because the reports from the existing literature make it quite clear that not all of our emotional experience can be accounted for by afferent facial feedback signals [6,9,10,33]. At the same time, however, these studies do show evidence supporting the facial feedback hypothesis; that at least some of our emotional experience is influenced by afferent signals from facial muscles.

Our data add further support to the facial feedback hypothesis by offering direct neural evidence. We chose to focus on the amygdala, which is known to be responsive to biologically relevant and socially salient stimuli, including facial expressions [34]. We specifically selected angry faces to probe the amygdala, since the BTX treatment in our study targeted the corrugator supercilii and the procerus – the glabellar muscles that we use when we frown and make angry faces. Prior to BTX treatment, our participants demonstrated greater amygdala activity to angry faces compared to happy faces. Similar activity of the amygdala was observed when effects of BTX wore off at least 9 months after its injection to the corrugator supercilii and the procerus. It was only during the period when BTX was active and the corrugator and procerus muscles were paralyzed that we did not see greater amygdala activity to angry vs. happy faces. This quadratic pattern of amygdala activity is consistent with the aforementioned behavioral and psychophysiological evidence in favor of the facial feedback hypothesis. It is clear from the data that the lack of afferent feedback signals from the corrugator supercilii and the procerus has impacted how the amygdala responds to angry vs. happy faces. The causal relationship we observe here is as follows: if there are no afferent feedback signals from the facial muscles to the brain, then amygdala activity to angry vs. happy faces is diminished. We believe that this means amygdala activity to angry vs. happy faces is, at least partially, relying on the feedback signals from the corrugator supercilii and the procerus. This implies that when we see an angry face, we detect and experience negatively valenced emotion by contracting and flexing the relevant muscles—the corrugator supercilii being the most prominent one in this case—and our amygdala uses these afferent signals to properly process the information.

Our data showing diminished activity of the amygdala due to BTX-induced paralysis of the corrugator/procerus are consistent with the findings reported by Hennenlotter and colleagues [24]. In their fMRI investigation, they also observed attenuated amygdala activity to angry faces when the participants were treated with BTX and had their corrugator paralyzed, compared to their placebo-injected controls [24]. There were slight differences as well—for instance, they found BTX-induced attenuation of amygdala activity only when the participants were asked to imitate the facial expressions that were shown on the screen, as opposed to just viewing them passively. In our study, participants were not explicitly asked to imitate the facial expressions they were viewing on the screen, but we observed diminished amygdala activity nonetheless. This discrepancy could stem from many factors, among which include differences in the experimental paradigm that was used to probe the amygdala. Since we did not explicitly instruct the participants to either imitate or not imitate the facial expressions, they might have been less aware of their facial muscular movements and thus less resistant to spontaneous elicitations of their own facial expressions when viewing other people’s faces. This may have also influenced the difference in the observed laterality of the amygdala—that is, whereas Hennenlotter and colleagues report that BTX affected the left amygdala [24], we found the right amygdala to be modulated by BTX-induced paralysis of the corrugator supercilii. Despite these relatively minor differences, we believe our data are in the same vein as the findings by Hennenlotter and colleagues, in the sense that both provide neural evidence supporting the facial feedback hypothesis [24]. Furthermore, an important contribution of the current study is the addition of a third post-BTX condition. As the last ‘A’ in the A-B-A design, this third condition allows us to directly test a causal relationship between facial feedback signals and amygdala activity and suggests that amygdala activation changes due to BTX injections are not permanent but rather reversible.

Another aspect of the current study that warrants discussion is the inclusion of happy faces in the analysis. Happy faces were used as a direct comparison to angry faces to gauge the level of amygdala activity—a common strategy that has been employed in a number of fMRI studies investigating the effects of negatively valenced facial expressions, such as anger or fear [35-37]. Upon closer examination of the data, our observations revealed that amygdala activity to happy faces actually showed an opposite quadratic pattern compared to angry faces. By subtracting amygdala activity to happy faces from angry faces and thus calculating amygdala activity to angry vs. happy faces, the U-shaped quadratic pattern became more prominent in our data. This inverted U-shaped pattern of activity to happy faces emerges as a topic of future inquiry, as BTX only had an effect on the corrugator supercilii and the procerus, not the zygomaticus major—the facial muscle we use to smile—but nevertheless had an impact on amygdala activity.

BTX-induced corrugator/procerus paralysis did not alter the participants’ perception of the valence of angry or happy faces. Regardless of BTX treatment, angry faces were consistently rated as being negatively valenced, and happy faces were consistently rated as being positively valenced. This was expected, as judging such clearly valenced facial expressions would not necessarily require feedback signals from the corrugator, given that the participants have prior experience and knowledge of the meaning of angry and happy faces. It is important to note that these behavioral data do not imply that afferent signals from the corrugator/procerus are not being used during processing of emotional facial expressions. Rather, these results hint at our ability to utilize multiple sources of information when making decisions on a given clearly valenced face, such that the lack of corrugator feedback signal is compensated by other factors including past experience and learned responses. Taking into account the EMG findings that show increased corrugator activity to angry facial expressions [15-17], negative pictures [18-20] and sounds [20], as well as other BTX-induced corrugator paralysis studies demonstrating behavioral effects such as impaired negative emotional language processing (i.e., significantly delayed reading times for sentences describing angry and sad situations) [21] and diminished subjective emotional experience in response to mildly positive video clips [10], it is clear that the feedback signals from the corrugator impact our emotional experiences, and it is beneficial to interpret our data within this context.

Limitations of the current study include the relatively small sample size. While within subject repeated measures (i.e., A-B-A design) mitigates this limitation to an extent, having a larger sample size, as well as a control group that is also cosmetically treated but without paralysis (see [10]) would strengthen the study. In addition, the present results might generalize only to females who would voluntarily report for BTX treatment. Next, it should be noted that the participants were exposed to surprised faces along with angry and happy faces during the experiment, and so the results reported here for happy and angry expressions could depend on the presence of surprised expressions in the experimental context. Finally, the current A-B-A design comprised uneven time intervals between each repeated measure, raising the possibility that the decrement in responses between pre-BTX and BTX (3–6 weeks) might be due to response habituation alone compared to the longer BTX and post-BTX interval (9 months). However, previous studies examining the test-retest reliability of amygdala BOLD activity to angry and fearful faces report reliable responsivity for both short (2 weeks; [38]) and long (1 year; [39]) time intervals.

## Conclusions

To summarize, our data support the facial feedback hypothesis by offering neural evidence concerning the causal relationship between activity in the corrugator/procerus muscles and in the amygdala. Using BTX to temporarily paralyze facial muscles, we have found that the amygdala closely tracks the state of the corrugator/procerus by displaying a quadratic pattern of activity. Specifically, amygdala responses to angry faces were diminished by BTX-induced corrugator/procerus paralysis but reverted to pre-BTX injections levels once the effect of the drug wore off. These findings offer preliminary causal evidence that amygdala activity is sensitive to facial feedback during the perception of the facial expressions of others.

## Abbreviations

BTX: botulinum toxin; fMRI: functional magnetic resonance imaging; EMG: electromyography; BOLD: blood-oxygen-level dependent; MNI: Montreal Neurological Institute; ANOVA: analysis of variance.

## Competing interests

The authors declare that they have no competing interests.

## Authors’ contributions

MJK, MN, FCD, EJR, and DD participated in the collection of the fMRI data and analyzed the data. MJK, PJW, TFH, and MAS conceived of the study, directed its design and coordination. MJK, PJW and MAS drafted the manuscript. All authors read and approved the manuscript.
